# QPromoters: sequence based prediction of promoter strength in *Saccharomyces cerevisiae*

**DOI:** 10.1080/26895293.2023.2168304

**Published:** 2023-01-20

**Authors:** Devang Haresh Liya, Mirudula Elanchezhian, Mukulika Pahari, Nithishwer Mouroug Anand, Shivani Suresh, Nivedha Balaji, Ashwin Kumar Jainarayanan

**Affiliations:** a Department of Physical Sciences, Indian Institute of Science Education and Research, Mohali, India; b Department of Biological Sciences, Indian Institute of Science Education and Research, Mohali, India; c Department of Computer Engineering, Ramrao Adik Institute of Technology, DY Patil Deemed to be University, Navi Mumbai, India; d Sheffield Institute for Translational Neuroscience (SITraN), University of Sheffield, Sheffield, UK; e School of Biology and Environmental Sciences (SBES), University College Dublin, Dublin, Ireland; f Kennedy Institute of Rheumatology, University of Oxford, Oxford, UK; g Interdisciplinary Bioscience Doctoral Training Program and Exeter College, University of Oxford, Oxford, UK

**Keywords:** Computational life sciences, bioinformatics and system biology

## Abstract

Promoters play a key role in influencing transcriptional regulation for fine-tuning the expression of genes. Heterologous promoter engineering has been a widely used concept to control the level of transcription in all model organisms. The strength of a promoter is mainly determined by its nucleotide composition. Many promoter libraries have been curated, but few have attempted to develop theoretical methods to predict the strength of promoters from their nucleotide sequence. Such theoretical methods are not only valuable in the design of promoters with specified strength but are also meaningful in understanding the mechanistic role of promoters in transcriptional regulation. In this study, we present a theoretical model to describe the relationship between promoter strength and nucleotide sequence in *Saccharomyces cerevisiae*. We infer from our analysis that the −49–10 sequence with respect to the Transcription Start Site represents the minimal region that can be used to predict promoter strength. https://qpromoters.com/ and a standalone tool https://github.com/DevangLiya/QPromoters to quickly quantify the strength of *Saccharomyces cerevisiae* promoters.

## Author summary

Regulating gene expression is a crucial aspect of metabolic engineering and synthetic biology. Promoter engineering plays a vital part in modulating transcriptional capacity and hence controlling gene expression. While there are tools to identify promoter regions in the eukaryotic genome, there are no simple tools to predict the strength of promoters in eukaryotes. Previous studies have shown that there exists a relationship between the promoter strength and the natural log of the promoter score. We use this relationship to identify the minimal promoter region in *Saccharomyces cerevisiae* that can be used to predict the strength of promoters. We have used a set of 18 standard promoters whose strengths were experimentally determined in previous studies to verify our model. We were able to classify promoters into three broad classes, namely weak, moderate, and strong, with high confidence. We have also developed a website and an open-source script that can be utilized to quantify promoter strength in *Saccharomyces cerevisiae* and streamline the process of promoter design.

## Introduction

*Saccharomyces cerevisiae* (*S. cerevisiae*), commonly known as brewer's yeast, is a widely used eukaryotic model organism in synthetic biology – it has applications in the production of biofuels, recombinant proteins and bulk chemicals (Nevoigt, 2008; Tang et al. 2020). Promoters are basic transcriptional elements that play a key role in manipulating genetic and metabolic pathways in *S. cerevisiae* by the regulation of protein expression both quantitatively and temporally (Scalcinati et al. 2012; Latimer et al. 2014) and are one of the most crucial component of yeast synthetic biology toolbox (Hubmann et al. 2014; Redden et al. 2015; Machens et al. 2017; Portela et al. 2017; Ottoz and Rudolf 2018; Decoene et al. 2019; Kotopka and Smolke 2020; Liu et al. 2020; Feng and Marchisio 2021). Promoters in *S. cerevisiae* have multiple components which together account for successful transcriptional regulation. The key components of a yeast promoter are an upstream activator sequence (UAS), an upstream repressor sequence (URS), a nucleosome-disfavoring sequence and a core promoter region.

**Figure 1.**
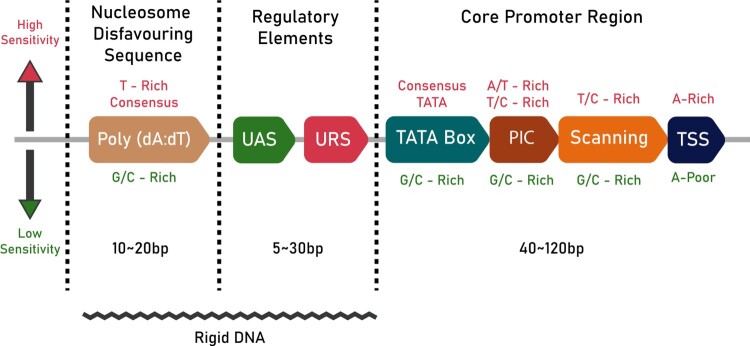
A schematic of promoter architecture in *S. cerevisiae*: The text in crimson (top) denotes the conditions necessary for high sensitivity, while the green text (bottom) denotes the conditions for lower sensitivity. The length of the different regions of the promoter are also given in bp. The jagged line at the bottom denotes the part of the promoter that is rigid in nature.

The core promoter is the DNA sequence nearest to the start codon, which interacts with RNA polymerase II (pol-II) and other general transcriptional factors to form the pre-initiation complex (PIC) (Tang et al. 2020). The core region also contains the TATA box, the transcription start site (TSS), a PIC localization stretch and a TSS scanning region for pol-II (Lubliner et al. 2013). The binding of general transcription factor proteins and histones to the TATA box facilitates the subsequent binding of pol-II, which along with several transcription factor proteins, constructs a transcription initiation complex that starts the mRNA synthesis from the TSS (Kanhere and Bansal 2005; Jiang and Pugh 2009). The nucleotide composition of different regions in the core promoter strongly influences the sensitivity of the promoter. Studies have shown that promoters with A/T – or T/C-rich PIC regions have higher sensitivities than promoters containing G/C-rich sequences (Lubliner et al. 2015). The position of the different regions of the core promoter is illustrated in Figure 1.

The UAS and URS are the regulatory components of a promoter and are located upstream to the core promoter region. UASs and URSs act as binding sites for transcription activators and repressors, respectively. The UAS enhances gene expression, provides additional stability, and plays a role in regulating the PIC formation process (West et al. 1984; Bitter et al. 1991). The UASs and URSs in *S. cerevisiae* are typically 10 bp long but can vary from 5 to 30 bp in length (Stewart et al. 2012).

The disfavoring nucleotide sequence is a stretch of DNA that decreases nucleosome occupancy to facilitate transcription (Struhl and Segal 2013). Poly(dA:dT) tract, a homopolymeric stretch of deoxyadenosine nucleotides, is a well-known nucleosome-disfavoring sequence commonly present in promoters (Workman 2006).

The structural properties of the promoter are vital for successful transcription. The flexibility of the promoter should be optimal to make sure that the binding sites are accessible and properly positioned to enable their recognition by transcriptional machinery (Jiang and Pugh 2009). In this regard, the bendability, or the propensity of each tri-nucleotide to bend, is essential (Kanhere and Bansal 2005). Existing studies indicate the presence of regions of low bendability about 100-200 bp upstream to the start codon (Miele et al. 2008) (illustrated in Figure 1 by a jagged line). Studies also indicate that the low bendability is caused by a combination of A/T richness and di- and tri-nucleotide composition (Akan and Deloukas 2008).

Promoters in *S. cerevisiae* can be either constitutive, that are relatively unaffected by internal and external signals and maintain stable levels of transcription, or inducible, which can initiate a drastic change in transcriptional levels in response to specific stimuli. These stimuli, called inducers, range from molecules such as metabolites, amino acids, and sugars to metal ions and environmental factors like pH and stress (Weinhandl et al. 2014; Gasser et al. 2015; Kim et al. 2015; Fischer et al. 2016; Rajkumar et al. 2016). Using endogenous promoters of *S. cerevisiae* for synthetic biology applications has disadvantages owing to an insufficiency of well-characterized promoters (Chen et al. 2018; Zhou et al. 2018). Thus, it is of utmost importance to characterize and quantify the strength of various *S. cerevisiae* promoters and create a database of the same.

Previous work has established a two-step approach for the quantitative prediction of the strength of promoters in *Escherichia coli* (*E. coli*), a prokaryotic model organism (Li and Zhang 2014; Bharanikumar et al. 2018). The linear relationship between the total promoter score and the promoter strength is well-established in *E. coli* (Berg and von Hippel 1987; Bharanikumar et al. 2018). In this study, we have presented a similar simplified model of the promoter strength in *S. cerevisiae* based on the promoter sequence.

We have done an extensive literature survey, and we observe that there are several established methods to quantify the strength of promoters (Rhodius Virgil and Mutalik Vivek 2010; Yada et al. 2011; Bharanikumar et al. 2018; Hayat et al. 2020; Zhao et al. 2020; Li et al. 2022; Zhao et al. 2022). We would like to highlight the fact that most of these methods deal with *E. coli* as the model organism. Our study is the only study that deals with the promoter sequences associated with eukaryotes to the best of our knowledge.

## Materials and methods

The core promoter sequences of 5117 promoters in *S. cerevisiae* were retrieved using the Sequence Retrieval in Tool Eukaryotic Promoter Database (EPD) (Dreos et al. 2017). The core promoter sequence consisted of −49–10 sequences with reference to the Transcription Start Site (TSS).

A Position Frequency Matrix (PFM) was generated from the motif of all 5117 promoter sequences. The PFM was then converted to Position Weight Matrix (PWM) or Position-Specific Scoring Matrix (PSSM) using the functions from biopython (Cock et al. 2009). The motif landscape was visualized using WebLogo (Crooks et al. 2004). The resulting PSSM was then used to calculate the ‘promoter score’ for all the promoters in the downstream analysis.

Taking inspiration from the well-established linear relationship between the total promoter score and the promoter strength in *E. coli* (Berg and von Hippel 1987), we modeled the promoter strength using a linear model with the promoter score as,

### Promoter strength = C0 + C1 × (Promoter score)

Lee et al*.* have characterized the strength of 19 constitutive promoters in *S. cerevisiae* using three fluorescence markers (Venus, mRuby2, and mTurquoise2) (Lee et al. 2015). We have used 18 of these promoter strengths in this study. The sequence for the promoter pREV1 was not found in EPD, due to which we have dropped it from our analysis. The normalized fluorescence values folded over the background were obtained from the authors of Lee et al. (2015). The log of these values gives the promoter strength. These were further divided by the strength of the strongest promoter (pTDH3) from Lee et al. (2015). We note that this step does not alter the linear relationship that is being tested but merely acts as a scaling. These values finally constitute the result space or the ‘Promoter strength’ in Eq. 1. We also have used another set of five promoters from Decoene et al. (2019), as the training set to further test the robustness of our model (Decoene et al. 2019). These fluorescence values were also subjected to the normalizations described above.

We define a ‘segment score’ which is simply the total score of a given segment of a given promoter as calculated from PSSM. This score is then divided by the highest score (corresponding to pTDH3) to obtain the feature space or ‘Promoter score’ part of the Eq. 1.

We then performed Ordinary Linear Regression (OLS) using the statsmodels package in python. C0 and C1 were left as free parameters to obtain the best fit. The quality of fit was then assessed using reduced r-squared and F-statistic. The significance of model parameters were assessed using the t-statistic. We also fitted a linear model to the residues to look for biases in the model. Scipy, statsmodels and Seaborn packages were used to perform, visualize, and test the linear regression (Seabold and Perktold 2010).

## Results and discussion

**Figure 2.**
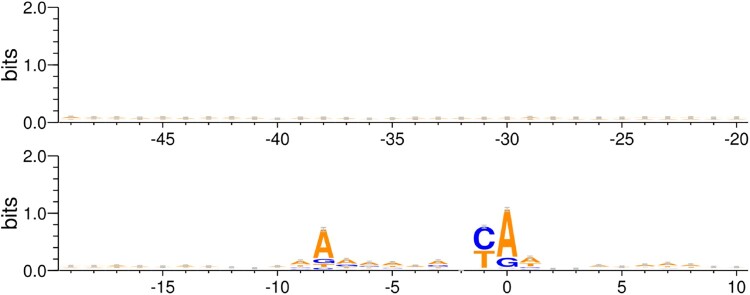
Motif of −49–10 region: Motif logo generated from all 5117 *S. cerevisiae* promoters from Eukaryotic Promoter Database. The motif is generated for −49–10 sequence with respect to the Transcription Start Site.

The 5117 native *S. cerevisiae* promoters from the EPD that were included in this study represent a diverse population of transcriptional regulators. Motif analysis on this set, shown in Figure 2, revealed that the promoters were diverse in terms of nucleotide composition. We see that the conservation along the entire promoter length is low, except for the TSS. Based on this, there are two possible regions or segments of the −49–10 sequence that can be considered for modeling the linear relationship in Eq. 1. 1) Highly conserved −9–1 segment, (2) −49 to X segment where X varies from −48 to 10. Results of modelling the promoter strength using these segments are discussed in the following sections along with their possible biological implications.

## −49 to X region

**Figure 3.**
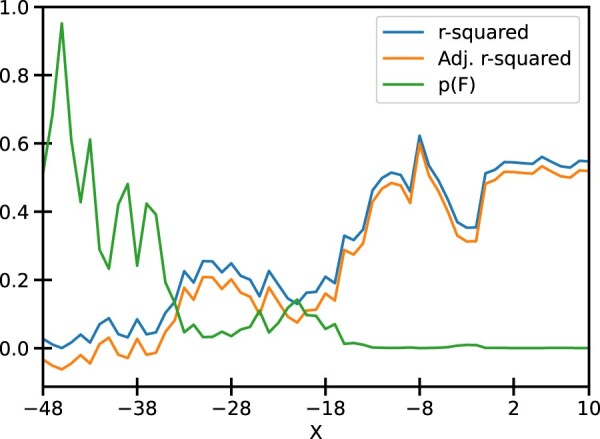
Various fit statistics for the linear regression of segment scores against the mRuby2 fluorescence: One of the ends of the promoter is fixed at −49 and nucleotides are added on the other end towards the TSS. The values of R-squared, Adj. R-squared, and *p*-value for F-statistic are tabulated in Table S1. Similar plots for Venus and mTurquoise2 fluorescence are given in Fig S2.

We sought to determine the shortest sequence that could model the relationship between experimental promoter strength and the segment scores. We first fix one end of such a segment at −49 position with respect to TSS and add nucleotides towards TSS until position ‘X’. Scores of the segments thus obtained were used to perform a linear regression described by the Eq. 1. The quality of fit estimators for each such regression is shown in Figure 3. It was observed that the quality of fit generally improves as more and more nucleotides are added. This is indicated by larger R-squared values and lower *p*-values for F-statistic. A saturation is reached at X = −1, after which adding more nucleotides does not improve the quality of fit by an appreciable amount, as shown in Figure 3 and S2).

**Figure 4.**
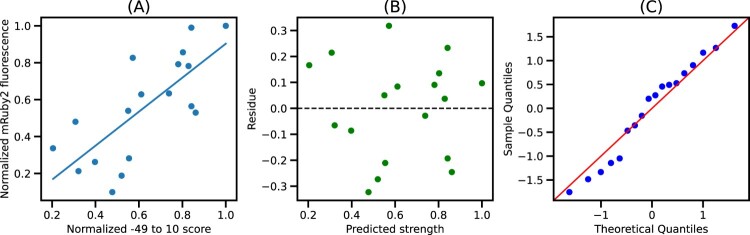
Best fit model for −49–10 segment: (A) Plot of normalized −49–10 score and normalized mRuby2 fluorescence along with the best fit model. (B) Residues obtained from the best fit model. (C) Quantile-Quantile plot of residues against normally distributed theoretical quantiles.

This saturation is sustained until X = 10, indicating that the quality of fit from −49 to −1 and −49–10 is mostly similar (Figures S4–S6). Since the −49–10 sequence was readily available in the EPD, further analysis focused on this 60 bp segment to ease the integration with the EPD. Figure 4 shows the plot of normalized −49–10 scores and normalized mRuby2 fluorescence along with the best fit model. We see that the residues for this model are randomly distributed around 0, indicating that the errors are uncorrelated, and the quantile-quantile plot shows that errors are normally distributed, thus validating all the assumptions for linear regression. Similar trends were observed using Venus and mTurquoise2 fluorescence as seen in Figures S2 and S3, respectively.

## −9–1 region

**Figure 5.**
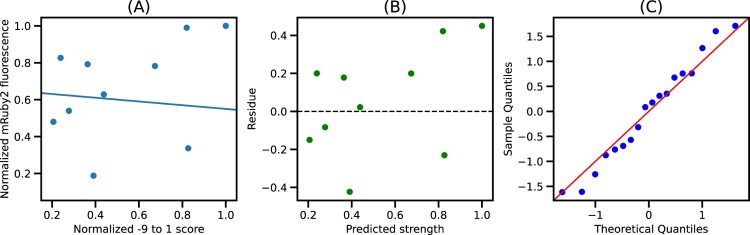
Best fit model for −9–1 segment: (A) Plot of normalized −9–1 score and normalized mRuby2 fluorescence along with the best fit model. (B) Residues obtained from the best fit model. (C) Quantile-Quantile plot of residues against normally distributed quantiles.

Motif analysis shown in Figure 2 showed that the −9–1 region to be the most conserved stretch. Previous work suggests that conserved sequences contribute significantly to the binding specificity of the promoter region (Berg and von Hippel 1987). As mentioned earlier, a high binding specificity is indicative of high promoter strength. Consequently, we sought to determine whether this hypothesis holds true for the promoters included in our analysis. The quality of fit observed in this case, however, is extremely poor as seen in the Figure 5. The *p*-value of F-statistic is 0.54 for regression using mRuby2 strengths. Therefore, we cannot reject the null hypothesis that there is no relationship between −9–1 score and promoter strength. This demonstrates that the above argument is not a dominant mechanism in defining the promoter strength.

## Values of model parameters

**Figure 6.**
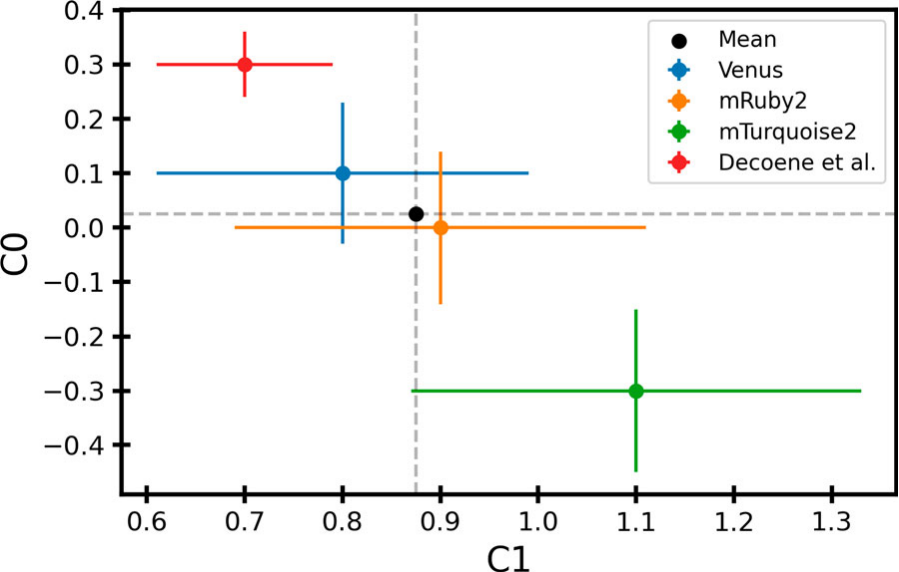
Best fit values of model parameters: The best fit values of C0 and C1 obtained using different fluorescence data as a proxy for promoter strength. The black dot shows the mean value of C0 and C1 weighted by error bars. The data corresponding to this plot can be found in Table S2.

We found that the value of intercept C0 in the model is close to zero for the best fit model and the value of slope C1 was between 0.8 and 1.1. Figure 6 shows the best fit values of C0 and C1 for different fluorescence along with their errors. Values of C0 and C1 obtained using mTorquoise2 fluorescence were slightly different from those obtained using Venus or mRuby2 fluorescence. These differences likely stem from the stochastic gene expression and noisy fluorescence signals in the experiments. However, they agree with each other within error bars. The value of C1 using the Decoene et al. (2019) dataset was within the 1-sigma error bars of Venus and mRuby2 fluorescence. The value of C0 differs significantly for this dataset, but we note that this is likely due to different experimental conditions and does not change the relative strengths of other promoters (Figures S7 and S8). The mean values of C0 and C1 are 0.025 and 0.87, respectively.

## QPromoters software

**Figure 7.**
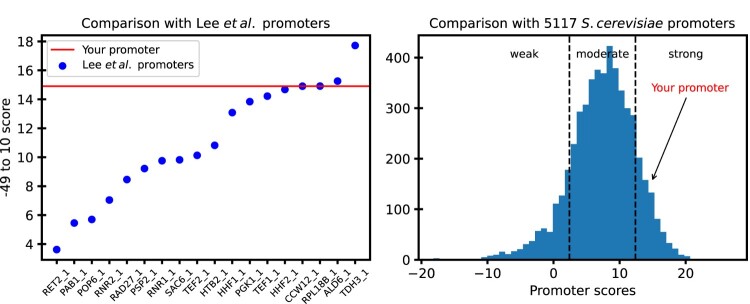
A sample output from QPromoters application: (A) −49–10 score of the user's promoter is shown as a horizontal line on a plot of scores of the characterized promoters from Lee et al. (2015) (B) Arrow shows the score of the user's promoter in reference to scores of all 5117 *S. cerevisiae* promoters in EPD.

We have developed an open-source, free-to-use standalone tool and a website to use our findings to predict the strength of *S. cerevisiae* promoters. The standalone tool can be found at https://github.com/DevangLiya/QPromoters, and the website can be found at https://qpromoters.com/. Users can either enter the −49–10 sequence of the engineered promoter or can retrieve this sequence directly from EPD by entering the EPDnew ID of the promoter. The tool then outputs the promoter score, promoter score normalized by pTDH3 score, promoter strength using the model described by equation Eq. 1, a plot showing the location of the user's promoter with reference to the 18 characterized promoters, and a histogram showing the location of the user's promoter with reference to all 5117 EPD promoters. An example of the figure returned by the program is shown in Figure 7.

## Conclusion

We analyzed the core promoter region of −49–10, with respect to TSS, to find a simple correlation between the score of this region and the experimental promoter activity. Analysis of the core promoter region revealed that there is a correlation between the promoter score and experimental promoter strength. Particularly, the −49–10 region of the core promoter was seen to be the best predictor of the promoter strength. We also observed a similar quality of fit between the −49 to −1 and −40–10 regions. The biological basis and significance of this sustained quality of fit needs further investigation. In addition to these findings, we have developed an open-source, free-to-use tool to predict the promoter strength of unknown promoters in *S. cerevisiae*.

Using computational tools to determine the essentiality of genes and strength of gene regulatory elements is of significant use in synthetic biology, as this tool will help in constructing recombinant circuits. This tool would also be helpful in experiments where fine-tuned regulation of gene expression is required and in studies involving transcription kinetics where characterizing promoter strength might be required. Consequently, these in-silico methods can precede and lower the risk of failure in *in vivo* experiments. Moreover, our web tool is useful in characterizing the strength of existing promoters in the EPD as well as predicting the strength of other engineered *S. cerevisiae* promoters on the basis of the promoter sequence.

## Supplementary Material

Supplemental Material

## Data Availability

The fluorescence data used in this work are openly available in the following publications: Lee et al. (2015) and Decoene et al. (2019) that issue datasets with DOIs. The standalone tool for predicting the promoter strength is open-source and available at https://github.com/DevangLiya/QPromoters. The online version of this tool can be accessed at https://qpromoters.com/.
