# Supplementary material for: QPromoters: sequence based prediction of promoter strength in Saccharomyces cerevisiae
Source: All Life. 2023 Jan 20;16(1):2168304. doi: 10.1080/26895293.2023.2168304 (PMC11478184; doi:10.1080/26895293.2023.2168304)
Supplement: Supplemental Material [file TFLS_A_2168304_SM9932.zip › Supplementary Material.docx]

**Supplementary Material**


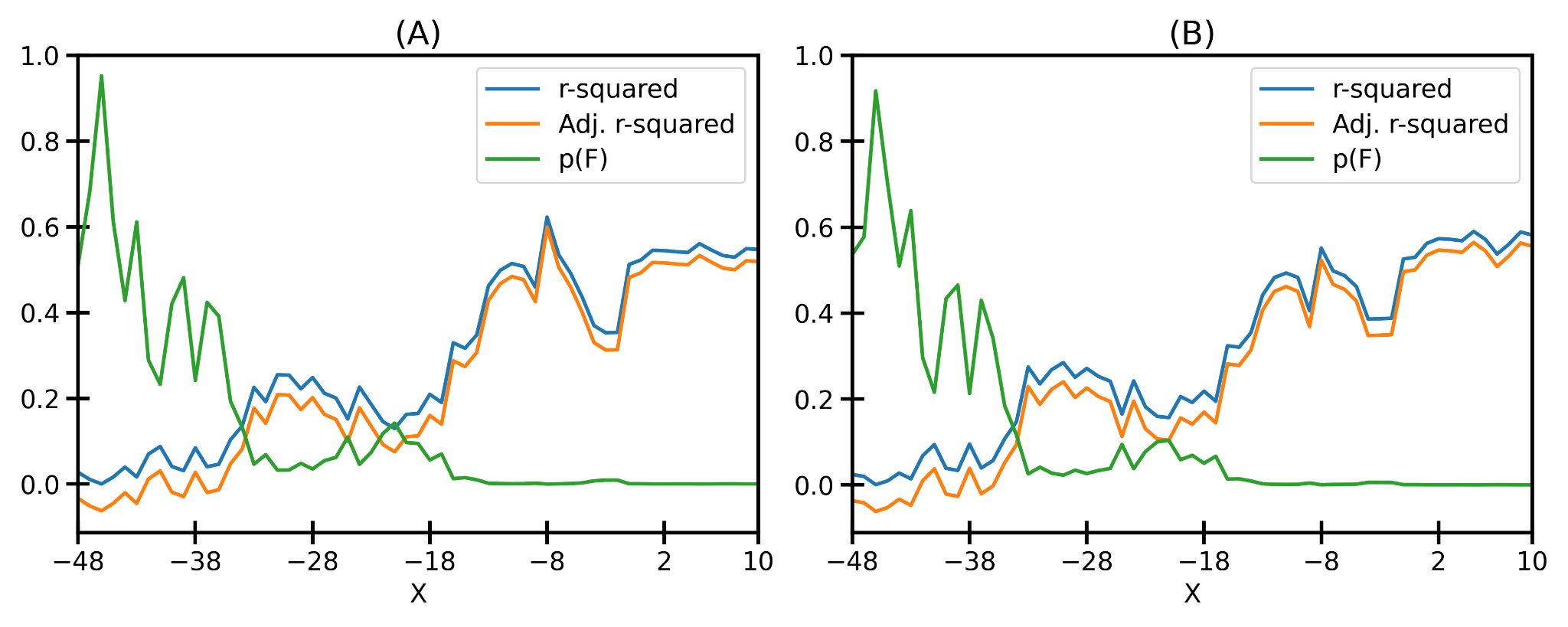


**Fig S1. Segment selection for Venus and mTurquoise2 fluorescence:** Various fit statistics for the linear regression of segment scores against the (A) Venus fluorescence and (B) against the mTurquoise2 fluorescence. One of the ends of the promoter is fixed at -49 and nucleotides are added on the other end towards TSS. The values of R-squared, Adj. R-squared, and p-value are tabulated in Table S1.


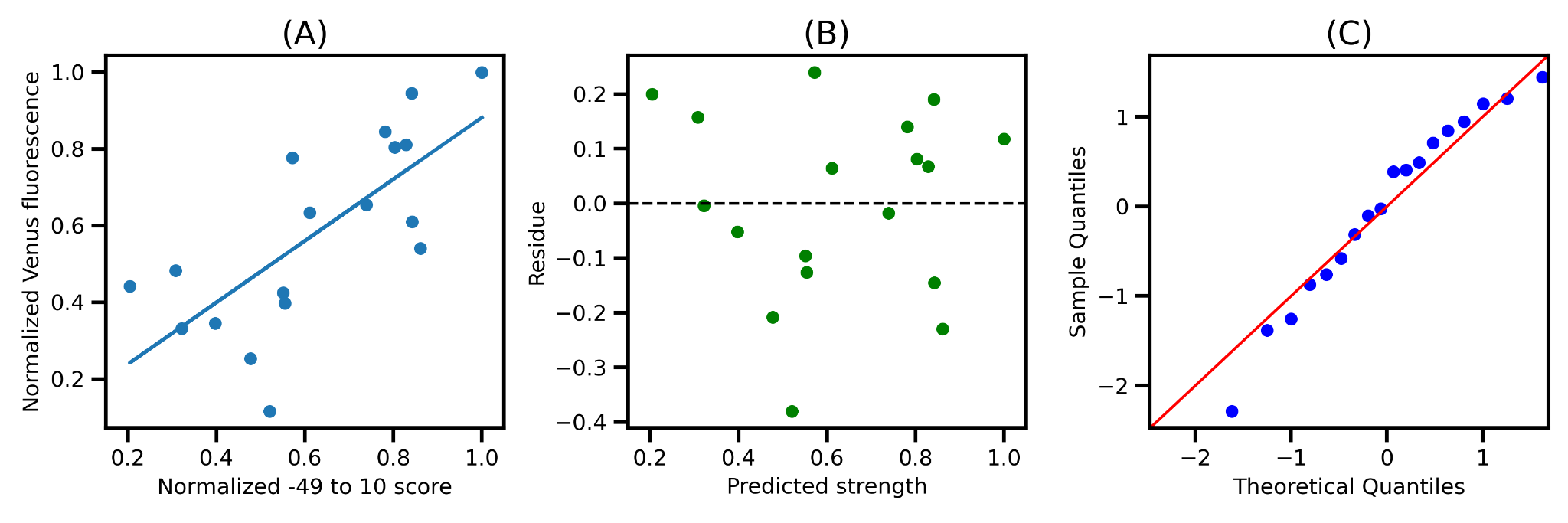


**Fig S2. -49 to 10 score against Venus fluorescence:** (A) Normalized promoter score is plotted against normalized Venus fluorescence. Blue line shows the best fit model. (B) Residues from the model. (C) Quantile-Quantile plot for normally distributed quantiles.


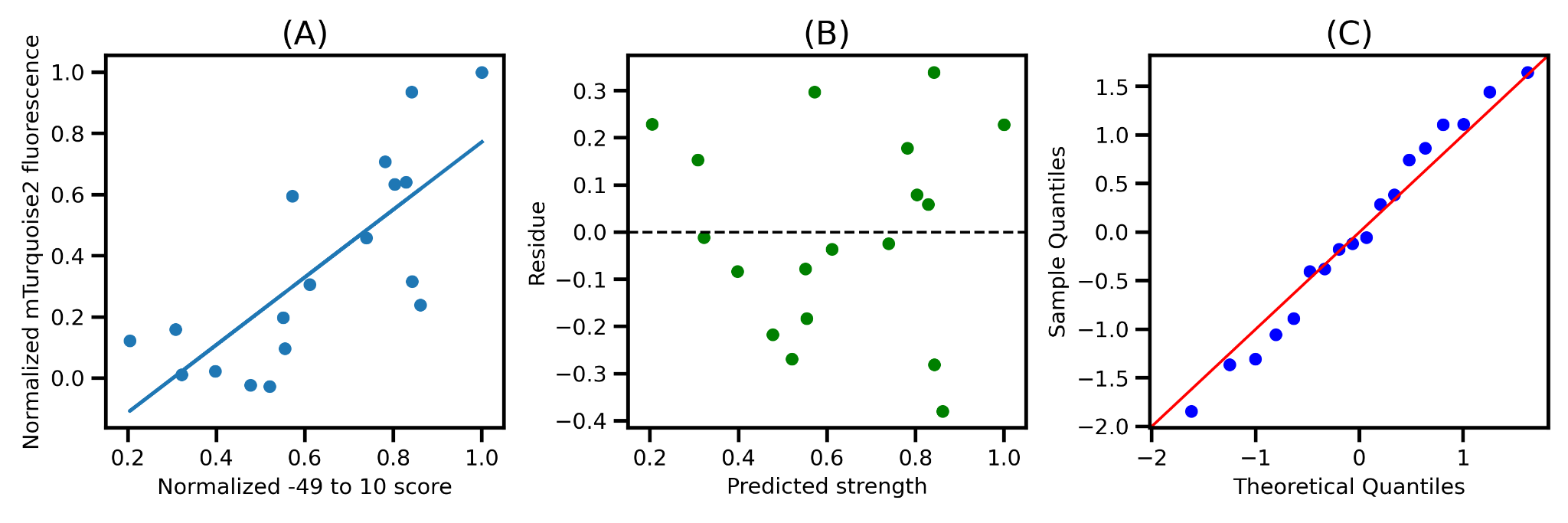


**Fig S3. -49 to 10 score against mTurquoise2 fluorescence:** (A) Normalized promoter score is plotted against normalized mTurquoise2 fluorescence. Blue line shows the best fit model. (B) Residues from the model. (C) Quantile-Quantile plot for normally distributed quantiles.


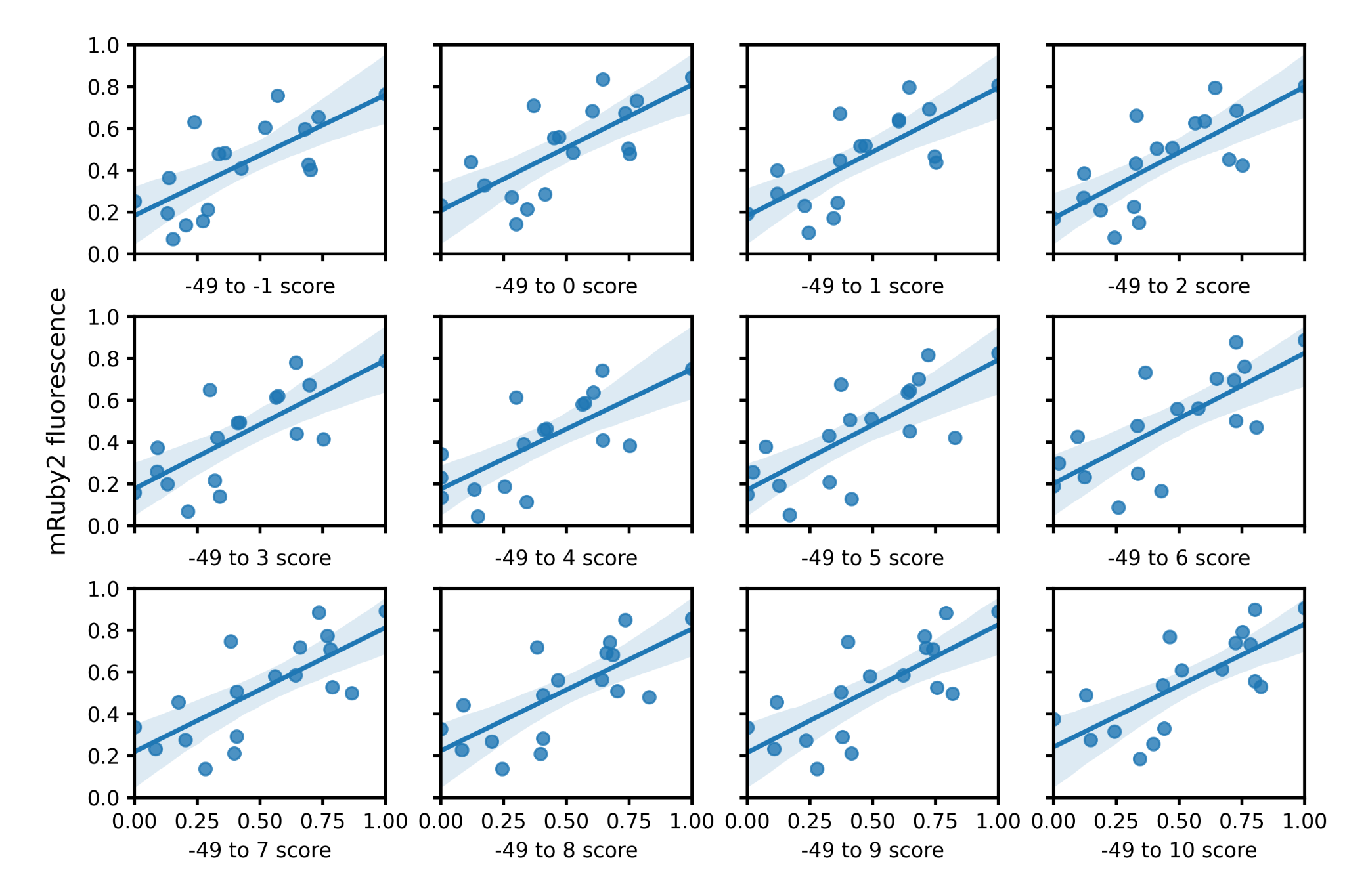


**Fig S4. -49 to X score against mRuby2 fluorescence:** Normalized mRuby2 fluorescence plotted against the normalized promoter score. The solid blue line shows the best fit line along with 95% interval. The promoter scores of different panels are calculated using only -49 to X region (where X varies from -1 to 10) with respect to TSS. See section 1 in results and discussion for more information.


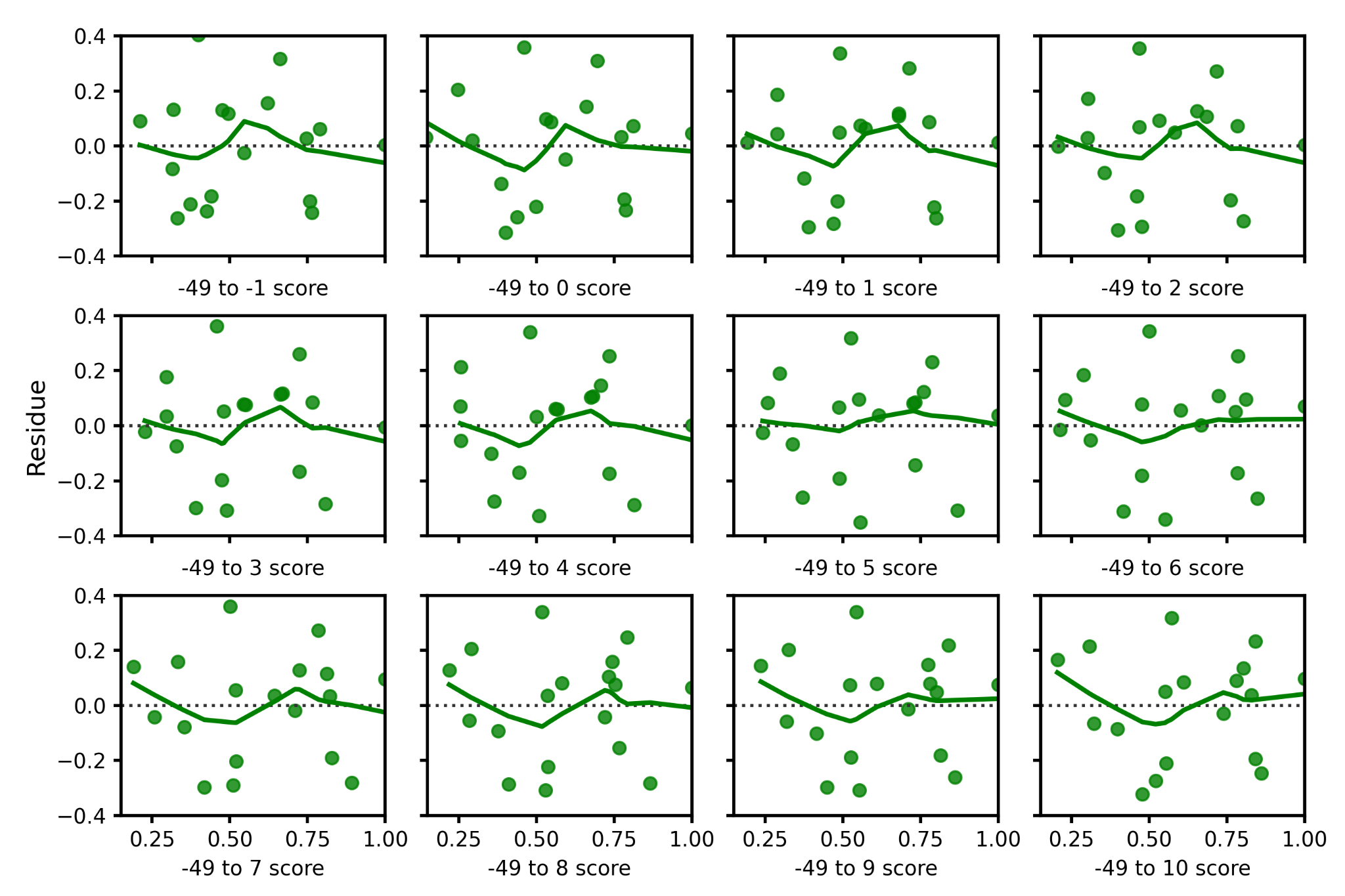


**Fig S5. Residues for -49 to X score against mRuby2 fluorescence:** This figure shows the residual plots corresponding to the fits in S4 Fig.


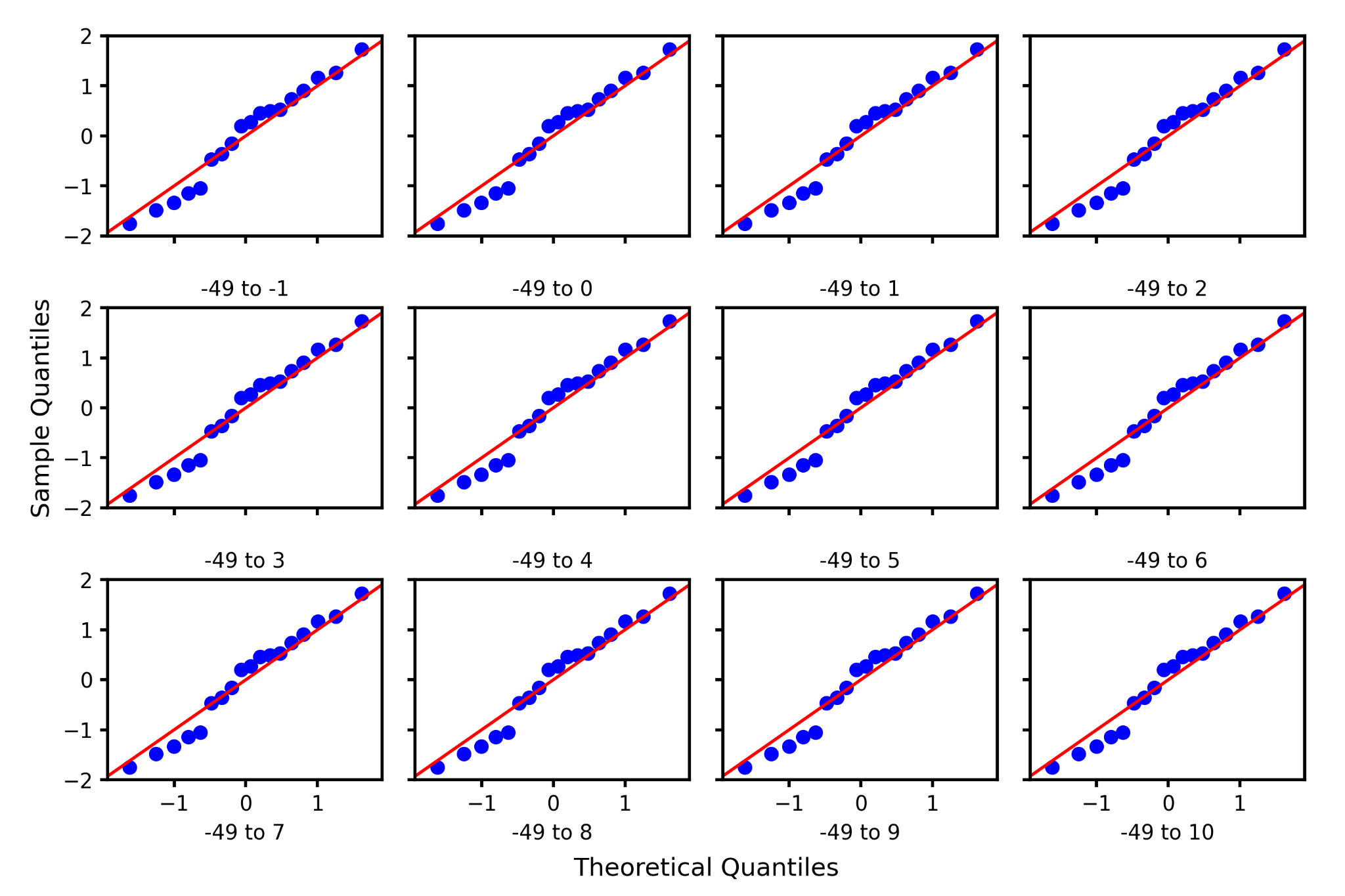


**Fig S6. Q-Q plot for -49 to X score against mRuby2 fluorescence:** This figure shows the Quantile-Quantile plots for residues corresponding to the fits in S4 Fig. plotted against normally distributed theoretical quantiles.


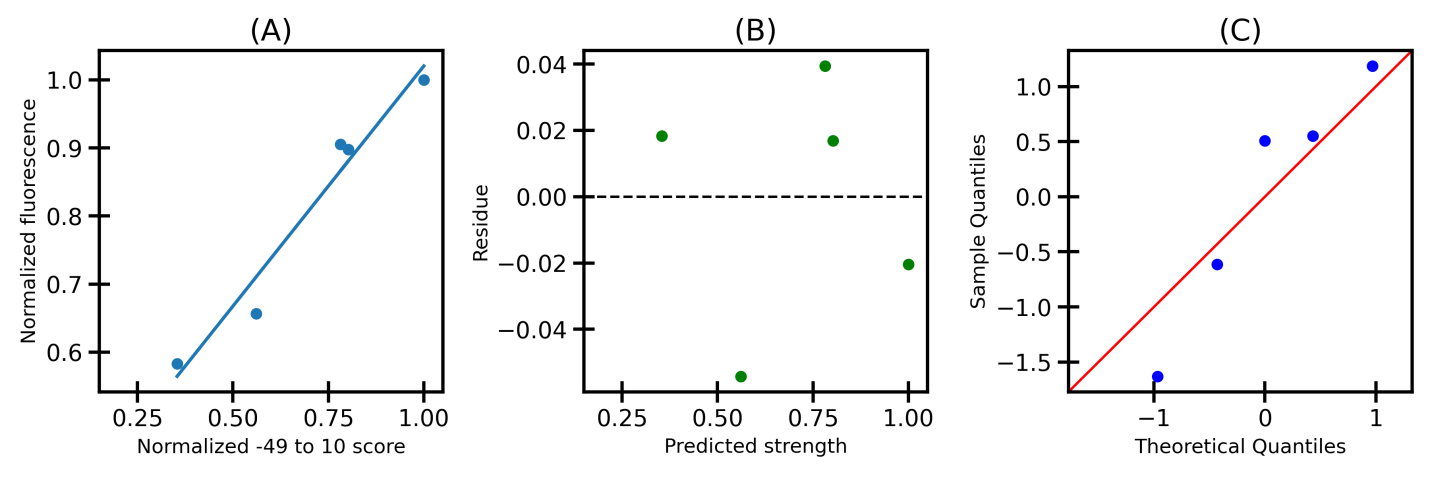


**Fig S7**: (A) Fit for normalized -49 to 10 promoter score and the normalized fluorescence values from Decoene et al., 2019. (B) Residues for the fit. (C) Quantile-Quantile plot for the fit.


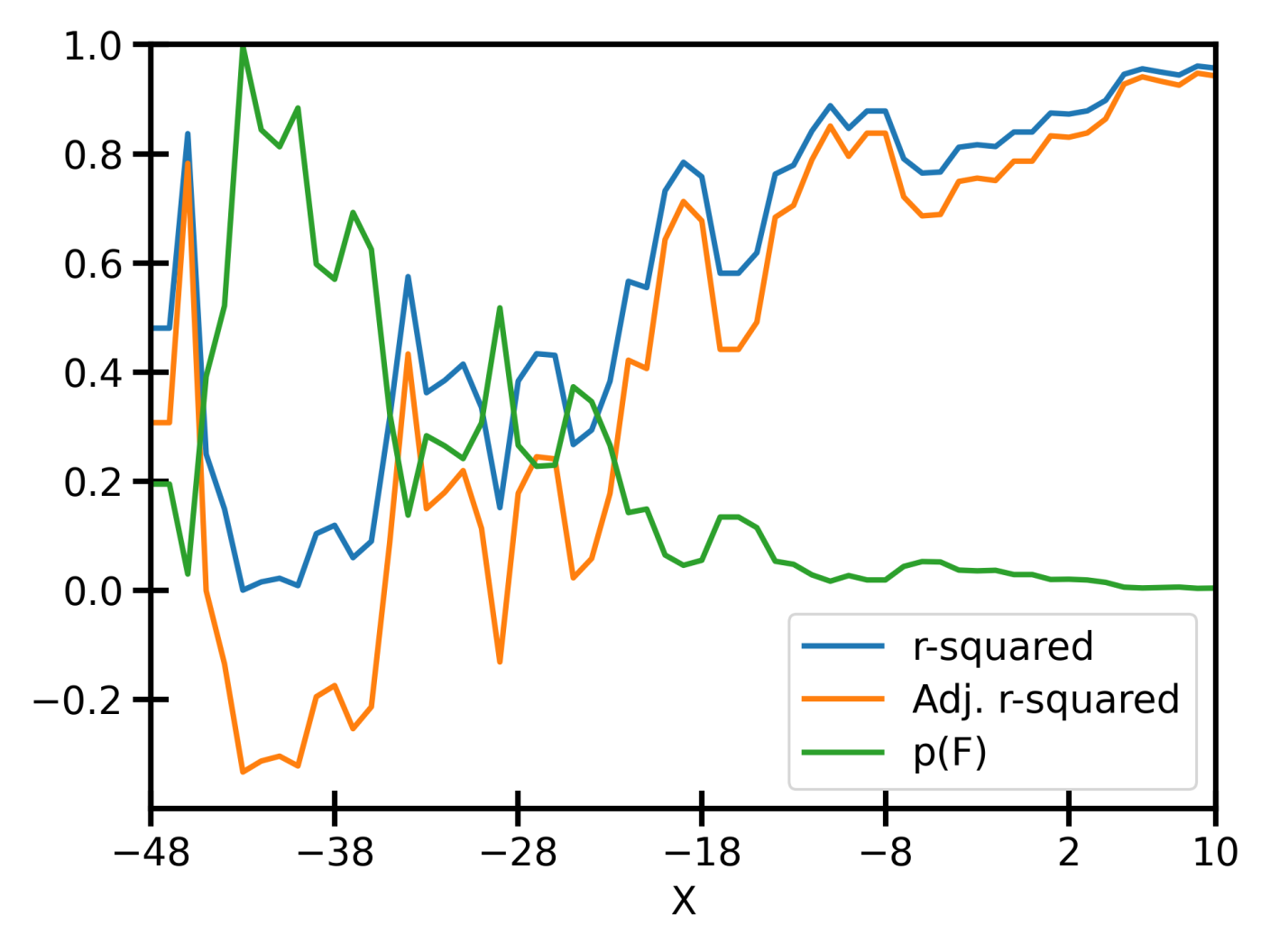


**Fig S8**: (A) Goodness-of-fit indicators for the fit between normalized -49 to 10 promoter score and the normalized fluorescence values from Decoene et al., 2019

| Region | R-squared | Adj. R-squared | p-value |
| --- | --- | --- | --- |
| -49 to -1 | 0.512 | 0.482 | 0.0008 |
| -49 to 0 | 0.523 | 0.493 | 0.0007 |
| -49 to 1 | 0.545 | 0.517 | 0.0005 |
| -49 to 2 | 0.545 | 0.516 | 0.0005 |
| -49 to 3 | 0.542 | 0.513 | 0.0005 |
| -49 to 4 | 0.54 | 0.511 | 0.0005 |
| -49 to 5 | 0.561 | 0.533 | 0.0003 |
| -49 to 6 | 0.546 | 0.518 | 0.0005 |
| -49 to 7 | 0.533 | 0.504 | 0.0006 |
| -49 to 8 | 0.529 | 0.5 | 0.0006 |
| -49 to 9 | 0.549 | 0.421 | 0.0004 |
| -49 to 10 | 0.547 | 0.519 | 0.0004 |

**Table S1. Assessment of the quality values and -49 to X scores:** Assessment of the quality of fit using mRuby2 fluorescence values and promoter score when one of the ends of the promoter is fixed at -49 and nucleotides are added on the other end.

| Parameter | vs. Venus | vs. mRuby2 | vs. mTurquoise2 | vs. Decoene et al. |
| --- | --- | --- | --- | --- |
| C0 | 0.1 ± 0.13 | 0.0 ± 0.14 | -0.3 ± 0.15 | 0.3 ± 0.06 |
| C1 | 0.8 ± 0.19 | 0.9 ± 0.21 | 1.1 ± 0.23 | 0.7 ± 0.09 |

**Table S2. Best fit values of model parameters using different fluorescence values as indicator for experimental promoter strength.**
