# Supplementary figures and images for: QPromoters: sequence based prediction of promoter strength in Saccharomyces cerevisiae
Source: All Life. 2023 Jan 20;16(1):2168304. doi: 10.1080/26895293.2023.2168304 (PMC11478184; doi:10.1080/26895293.2023.2168304)

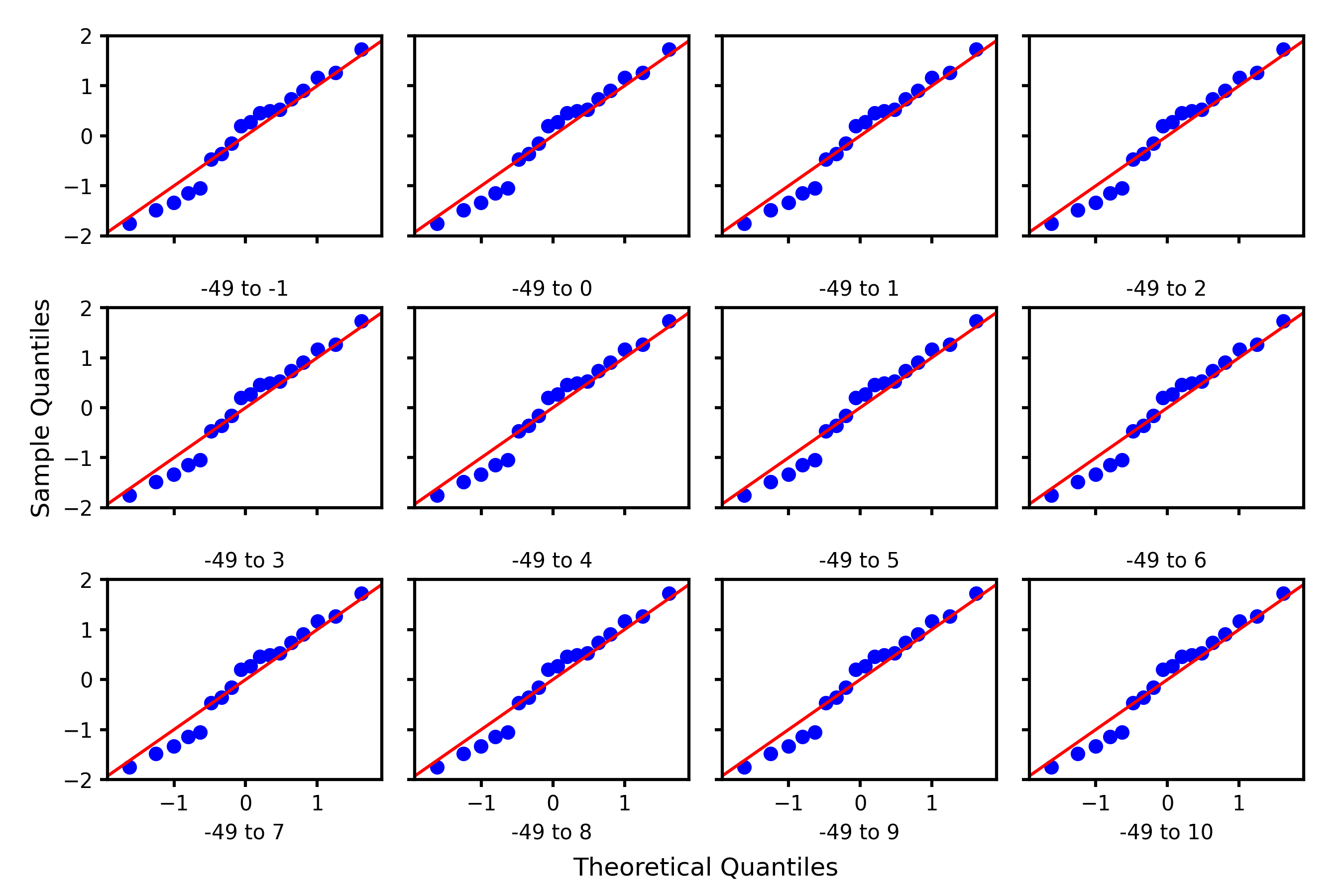

Supplement: Supplemental Material [file TFLS_A_2168304_SM9932.zip › Supplementary_figure_S6.png]

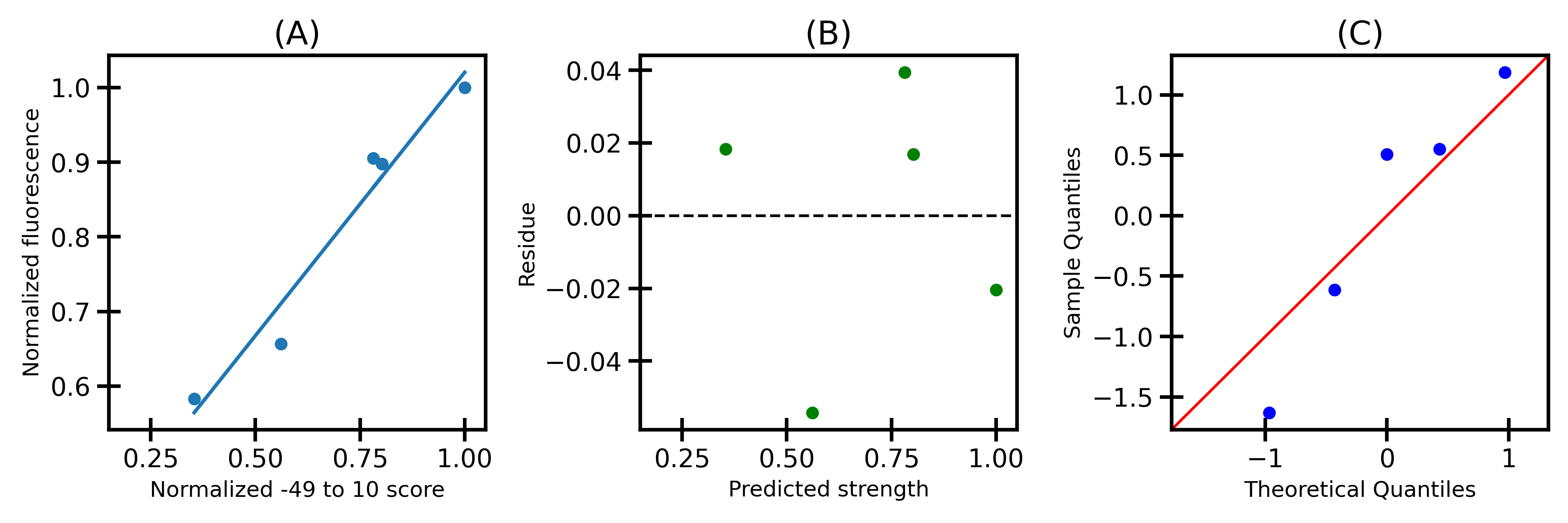

Supplement: Supplemental Material [file TFLS_A_2168304_SM9932.zip › Fig S7.png]

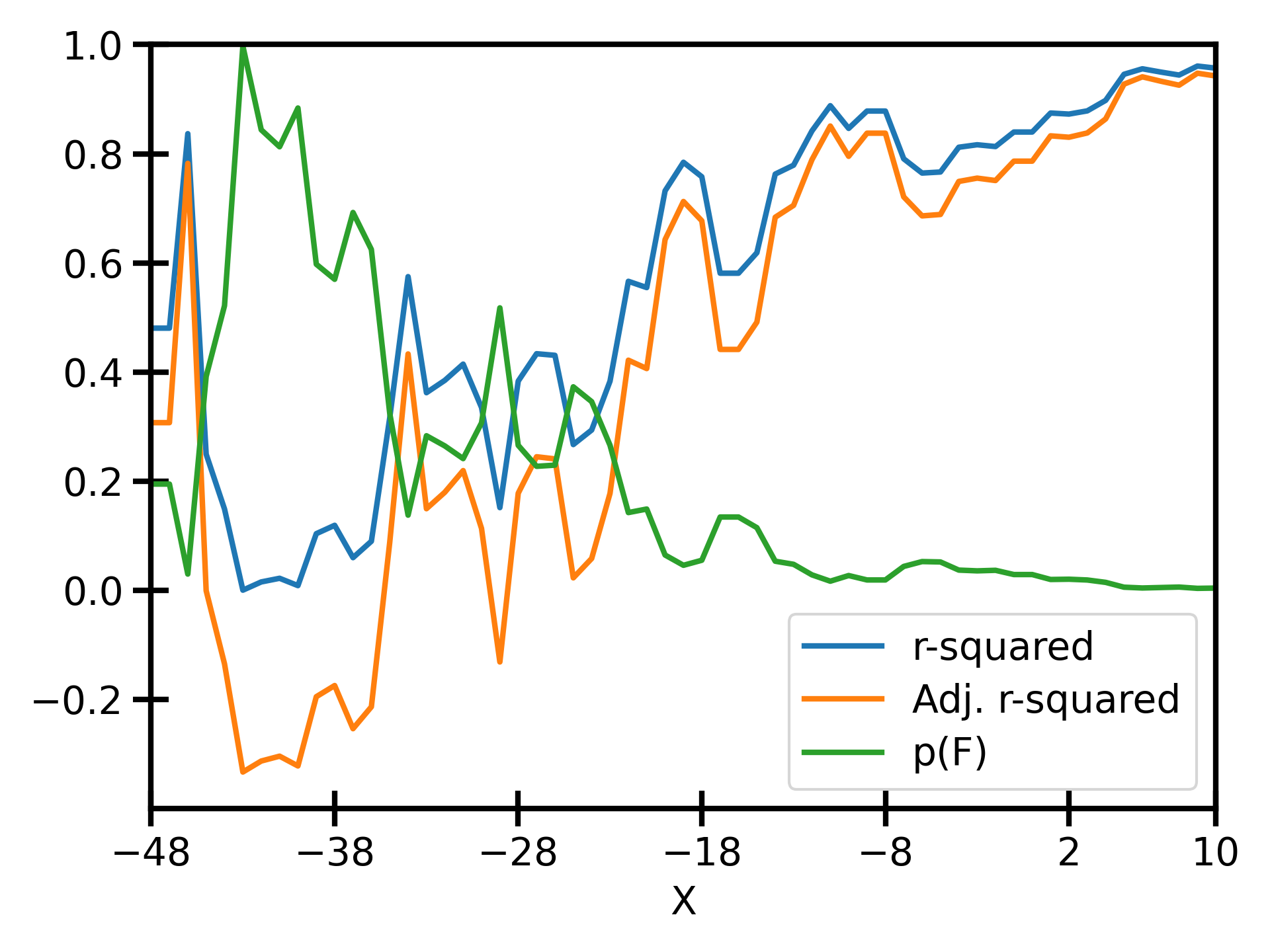

Supplement: Supplemental Material [file TFLS_A_2168304_SM9932.zip › Fig S8.png]

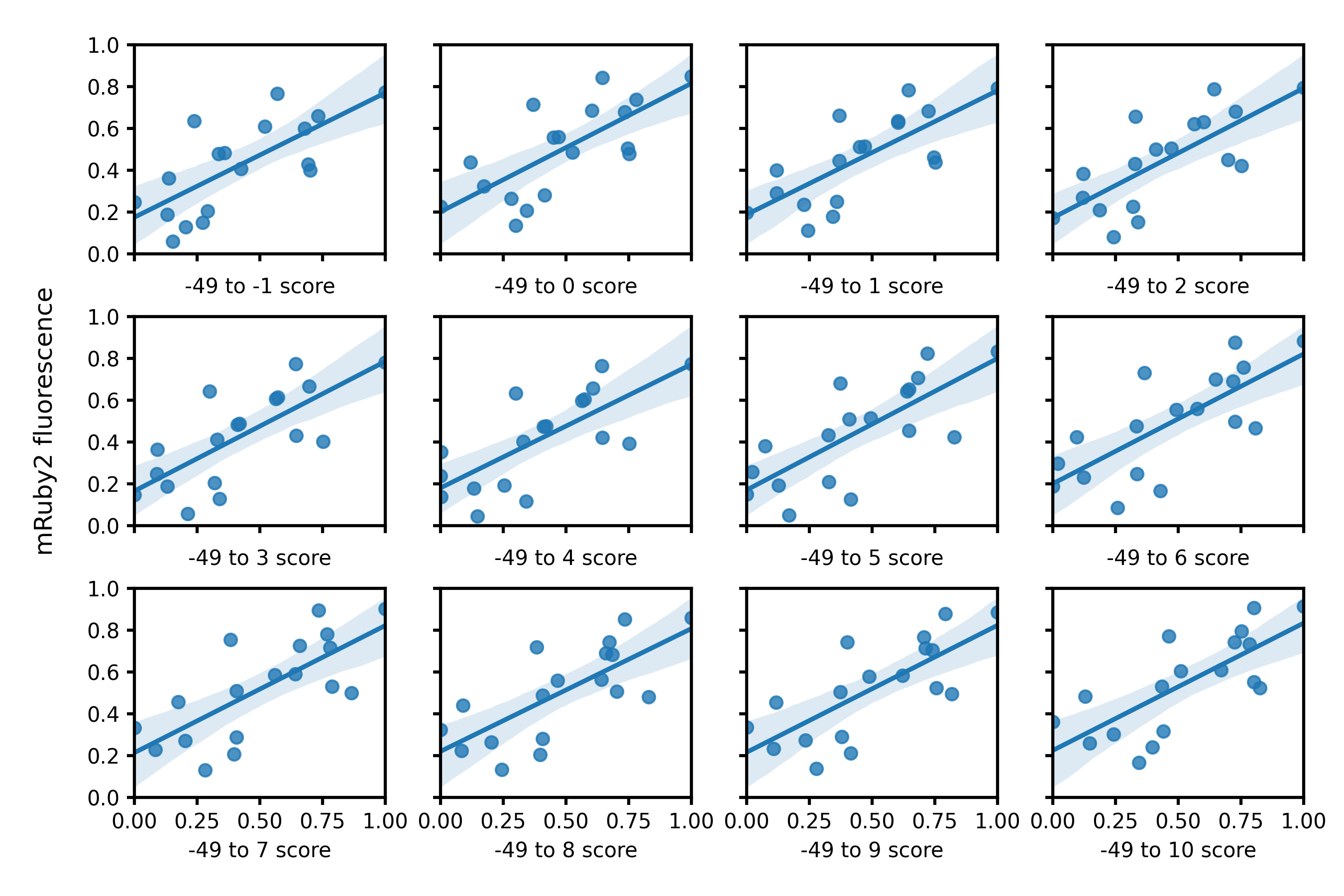

Supplement: Supplemental Material [file TFLS_A_2168304_SM9932.zip › figS4.tif]

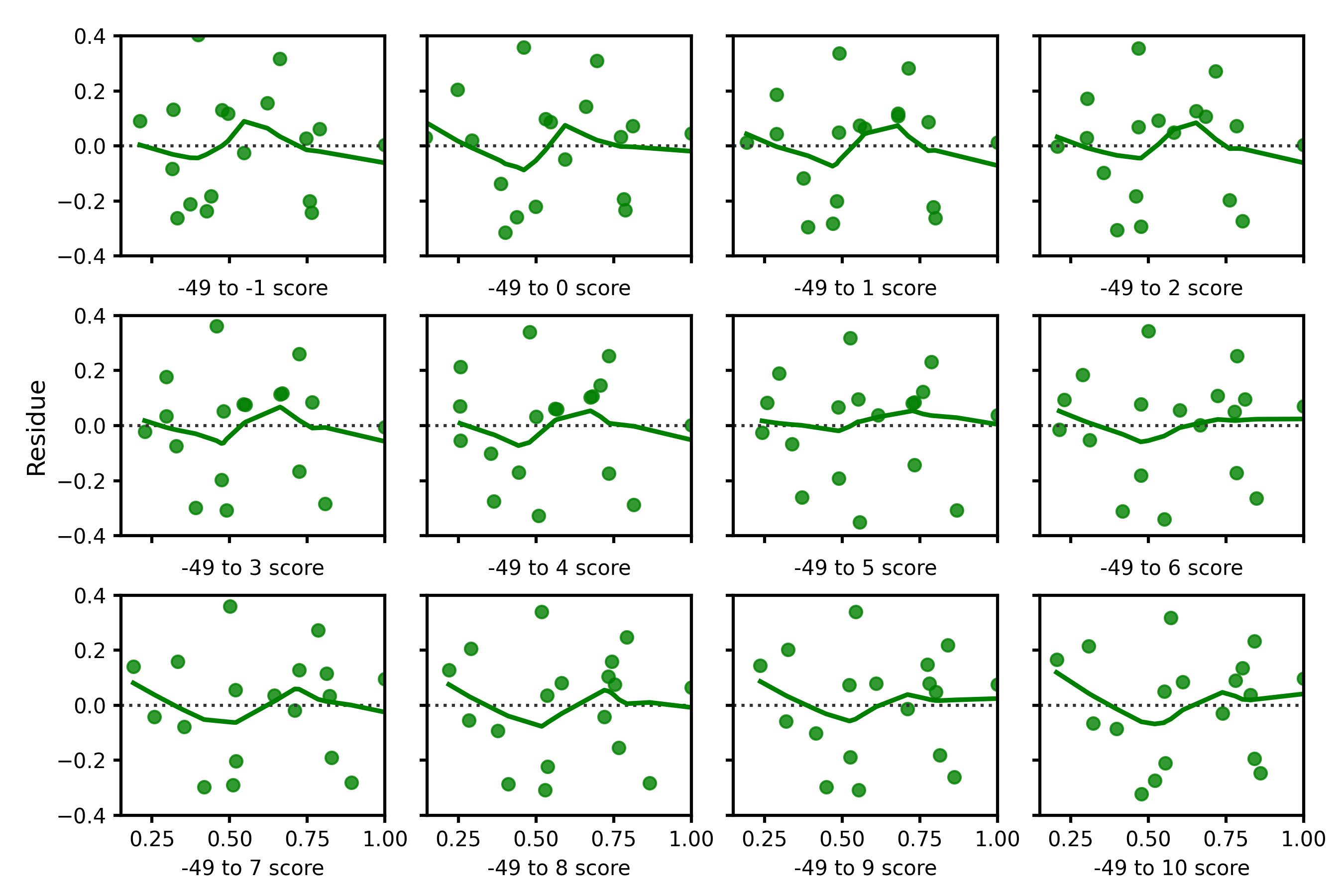

Supplement: Supplemental Material [file TFLS_A_2168304_SM9932.zip › figS5.tif]
